# Supplementary material for: GABAergic inhibition in dual-transmission cholinergic and GABAergic striatal interneurons is abolished in Parkinson disease
Source: Nat Commun. 2018 Apr 12;9:1422. doi: 10.1038/s41467-018-03802-y (PMC5897332; doi:10.1038/s41467-018-03802-y)
Supplement: Supplementary file 1 — Supplementary Information [file 41467_2018_3802_MOESM1_ESM.pdf]

Supplementary materials

GABAergic inhibition in dual-transmission cholinergic and GABAergic striatal interneurons is abolished in Parkinson disease

N. Lozovaya et al

Supplementary Table 1

Statistical analysis (significant differences are highlighted in blue)

Morphological metrics and Sholl analysis on dendrites
One-way ANOVA Kruskal-Wallis test with Dunn's Multiple Comparison post-hoc test.
Fig. 1d and 3h

**Dendrites**	Control CGIN n = 7 cells N = 5 mice Mean ± SEM	Control CIN n = 7 cells N = 5 mice Mean ± SEM	6-OHDA CGIN n = 8 cells N = 5 mice Mean ± SEM	Control CGIN vs control CIN Corrected P value	Control CGIN vs 6-OHDA CGIN Corrected P value
Number of trunks	3.3 ± 0.3	4.9 ± 0.3	5.0 ± 0.3	0.010	0.003
Number of nodes	21.3 ± 3.2	34.7 ± 5.4	40.4 ± 3.3	0.092	0.003
Number of tips	24.4 ± 3.1	39.6 ± 5.5	45.4 ± 3.3	0.071	0.002
Total length (µm)	2607 ±393	4495 ± 559	4722 ± 370	0.042	0.005
Enclosing radius (µm)	245.1 ± 28.0	228.9 ± 16.1	281.1 ± 15.0	0.999	0.182
Critical radius (µm)	78.8 ± 10.1	89.8 ± 6.1	80.6 ± 5.2	0.336	0.989
Critical value	13.7 ± 1.7	25.1 ± 3.2	22.5 ± 2.1	0.013	0.024

Sholl analysis on dendrites – Kolmogorov-Smirnov test
Fig. 1e and 3i

Groups	P value
Control CGIN vs control CIN	0.0001
Control CGIN vs 6-OHDA CGIN	0.0001

Supplementary Table 2

Statistical analysis (significant differences are highlighted in blue)

Spontaneous spiking frequency of control CGIN, CIN and PLTS interneurons in cell-attached recordings Fig. 1h

One-way ANOVA with Fisher's LSD post-hoc test

Groups	n (cells)	N (mice)	Mean ± SEM (Hz)
CGIN	23	8	2.63 ± 0.03
CIN	8	4	4.13 ± 0.07
PLTS	13	4	9.96 ± 0.08

groups	Nb of spikes	Nb of spikes	Univariate ANOVA between groups	Post-hoc test between the groups Fisher test
CGIN vs CIN	3905	6180		$7*10^{-54}$
CGIN vs PLTS	3905	3605		0
CIN vs PLTS	6180	3605	0	0

Supplementary Table 3

Statistical analysis (significant differences are highlighted in blue)

Intrinsic properties of CGIN, PLTS, CIN and FS

Supplementary Fig. 3

			Firing properties					
Groups	n	N	AHP amplitude (mV)	SAG amplitude (mV)	Input resistance (MΩ)	Rheobase (mV)	AP duration (ms)	Maximal frequency (Hz)
PLTS	11	5	4.2 ± 0.4	0.86 ± 0.03	559.1 ± 36.9	17.3 ± 2.0	1.51 ± 0.11	29.5 ± 2.0
FS	13	5	12.1 ± 0.8	0.99 ± 0.01	149.1 ± 20.3	147.8 ± 13.8	0.67 ± 0.05	N/A
CGIN	18	8	10.7 ± 1.0	0.73 ±0.01	207.3 ± 13.4	22.2 ± 3.4	2.60 ± 0.12	8.7 ± 1.1
CIN	11	4	11.8 ± 1.0	0.68 ± 0.02	260.7 ± 25.6	15.0 ± 2.7	2.49 ± 0.11	13.3 ± 2.1

One-way ANOVA with Fisher's LSD post-hoc test

Intrinsic properties	Groups	Univariate ANOVA between the groups	Post-hoc test between the groups Fisher test
AHP	PLTS *vs* CGIN		$4*10^{-6}$
	CIN *vs* CGIN		0.1
	CIN *vs* PLTS		$3*10^{-6}$
	FS *vs* CGIN		0.2
	FS *vs* PLTS		$4*10^{-7}$
	FS *vs* CIN	$7*10^{-7}$	0.83
SAG	PLTS *vs* CGIN		$1.7*10^{-7}$
	CIN *vs* CGIN		0.046
	CIN *vs* PLTS		$2*10^{-9}$
	FS *vs* CGIN		$1*10^{-16}$
	FS *vs* PLTS		$3*10^{-6}$
	FS *vs* CIN	$2*10^{-17}$	$2*10^{-17}$
Input resistance	PLTS *vs* CGIN		$2*10^{-14}$
	CIN *vs* CGIN		0.10
	CIN *vs* PLTS		$6*10^{-11}$
	FS *vs* CGIN		0.07
	FS *vs* PLTS		$1.14*10^{-15}$
	FS *vs* CIN	$1*10^{-15}$	0.002
Rheobase	PLTS *vs* CGIN		0.52
	CIN *vs* CGIN		0.37
	CIN *vs* PLTS		0.79
	FS *vs* CGIN	$5*10^{-19}$	$4*10^{-19}$

	FS *vs* PLTS		$4*10^{-18}$
	FS *vs* CIN		$4*10^{-18}$
AP duration	PLTS *vs* CGIN		$1*10^{-9}$
	CIN *vs* CGIN		0.47
	CIN *vs* PLTS		$1*10^{-6}$
	FS *vs* CGIN		$6.5*10^{-18}$
	FS *vs* PLTS		$9*10^{-6}$
	FS *vs* CIN	6.54E-18	$7*10^{-15}$
Maximal frequency	PLTS *vs* CGIN		$1.8*10^{-11}$
	CIN *vs* CGIN		0.046
	CIN *vs* PLTS	8.41E-11	$1.3*10^{-7}$

Supplementary Table 4

Statistical analysis (significant differences are highlighted in blue)

Pause-response and rebound in CGIN vs CIN:

Spike count at different time windows (0-400 ms after train and 800-1000 ms after train) normalized to spikes count before train. Cell-attached recordings

One-way ANOVA with Fisher's LSD post-hoc test

Figure 1m

groups	n (cell)	N (mice)	Mean ± SEM
CGIN before train	6	4	1.00 ± 0.04
CIN before train	6	3	1.00 ± 0.04
CGIN 400 ms after train	6	4	0.04 ± 0.03
CIN 400 ms after train	6	3	0.53 ± 0.13
CGIN 1000 ms after train	6	4	2.54 ± 0.29
CIN 1000 ms after train	6	3	0.63 ± 0.09

Groups	n1	n2	Univariate ANOVA between the groups	Post-hoc test between the groups Fisher test
CGIN before train vs CGIN 400 ms after train	6	6		$2*10^{-5}$
CIN before train vs CIN 400 ms after train	6	6		$5*10^{-4}$
CGIN before train vs CGIN 1000 ms after train	6	6		$2*10^{-9}$
CIN before train vs CIN 1000 ms after train	6	6		0.11
CGIN 400 ms after train vs CIN 400 ms after train	6	6		$8*10^{-12}$
CGIN 1000 ms after train vs CIN 1000 ms after train	6	6	$1*^{-15}$	$5*10^{-10}$

Supplementary Table 5

Statistical analysis (significant differences are highlighted in blue)

Effects of isoguvacine on spontaneous spiking frequency of CGINs and CINs in control animals (cell-attached recordings) normalized to control.

Supplementary Fig. 5b

Groups	n (cells)	N (mice)	Mean ± SEM	Paired/2 samples sample 2-tailed t-test
CGIN	13	7	0.42 ± 0.08	1*10-5
CIN	6	3	0.83 ± 0.10	ns
CGIN vs CIN				0.008

Supplementary Table 6

Quantification of ChAT and VGAT markers.
Fig. 2b

	N (mice)		Sum	Mean ± SEM
	Wt	Lhx6-GFP		
Number of $ChAT^+$ cells	4	4	381	47.6 ± 6.8
Number of $ChAT^+$ $VGAT^+$ cells	4	4	224	28.0 ± 3.5
Number of $ChAT^+$ $VGAT^+$ GFP^+ cells	0	4	98	24.5 ± 4.2
% of $ChAT^+$ $VGAT^+$ cells	4	4	-	58.8 ± 5.0
% of $ChAT^+$ $VGAT^+$ cells that express GFP	0	4	-	95.1 ± 1.8

Supplementary Table 7

iDISCO counting
Figure 2c

	Number of $ChAT^+$ cells	Number of $ChAT^+$ $EGFP^+$ cells	% of $ChAT^+$ $EGFP^+$ cells
N (mice)	3	3	3
Median	2160	933	55
Mean± SEM	1873 ± 324	978 ± 191	52.7 ± 5.8

Supplementary Table 8

Quantification of $ChAT^+$ and YFP^+ cells in ChAT-ChR2-$EYFP^{+/-}$ mice.
Supplementary Fig. 7

	N (mice)	N (neurons)	Mean ± SEM
Number of $ChAT^+$ cells	2	499	250 ± 13.5
Number of YFP^+ cells	2	488	244 ± 12
Number of $ChAT^+$ YFP^+ cells	2	477	238.5 ± 17.68
% of YFP^+ $ChAT^+$ cells compared to the total number of YFP^+ cells	2	-	97,73 ± 0.32

Supplementary Table 9
Quantification of $ChAT^+$ and YFP^+ cells in ChAT-ChR2-EYFPxLhx6-iCre; AI14-tomato mice.

	N (mice)	N(slices)	N (neurons)	Mean ± SEM
Number of YFP^+ cells	2	7	217	32.4 ± 4.33
Number of $ChAT^+$ cells	2	7	227	31.0 ± 3
Number of $ChAT^+$ YFP^+ cells	2	7	217	31.0 ± 8.2
% of YFP^+ $ChAT^+$ cells compared to the total number of YFP^+ cells	2	7	-	95,2 ± 2.46
% of YFP^+ $ChAT^-$ cells compared to the total number of YFP^+ cells	2	7		4.8 ± 2.5

Supplementary Table 10

Statistical analysis (significant differences are highlighted in blue)

DF_{GABA} (mV) inferred from single $GABA_A$ channels recordings

Figure 4c

Groups	n (cells)	N (mice)	Mean ± SEM	2 samples 2-tailed t-test
Control mice	17	5	-3.04±1.05	$3*10^{-9}$
6-OHDA mice	12	4	7.99 ± 0.43	

Supplementary Table 11

Resting potential (E_m, mV) inferred from single NMDA channels recordings

Figure 4f

Groups	n (cells)	N (mice)	Mean ± SEM	2 samples 2-tailed t-test
Control mice	7	3	-63.03±3.51	1
6-OHDA mice	7	3	-65.90± 2.30	

Supplementary Table 12

Statistical analysis (significant differences are highlighted in blue)
Effects of isoguvacine on CGIN spontaneous spiking frequency (cell-attached recordings) normalized to control.
One-way ANOVA with Fisher's LSD post-hoc test
Figure 4j

Groups	n (cells)	N (mice)	Mean ± SEM	Paired sample 2-tailed t-test
Control mice	13	7	0.42 ± 0.08	$1*10^{-5}$
6-OHDA mice	16	8	1.15 ± 0.07	0.05565
Bumetanide-treated 6-OHDA mice	13	3	0.48 ± 0.07	$5*10^{-6}$

Groups	n1	n2	Univariate ANOVA between groups	Post-hoc test between groups Fisher test
Control mice vs 6-OHDA mice	13	16	$4.7*10^{-9}$	$1.3*10^{-8}$
6-OHDA mice vs Bumetanide-treated 6-OHDA mice	16	13		$8.8*10^{-8}$

Supplementary Table 13

Statistical analysis (significant differences are highlighted in blue)
Effects of isoguvacine on spontaneous spiking frequency of CGIN in sham-operated and control animals (cell-attached recordings) normalized to control.
Supplementary Fig. 13a

Groups	n (cells)	N (mice)	Mean ± SEM	Paired/2 samples sample 2-tailed t-test
Sham mice	9	3	0.31 ± 0.07	$8*10^{-6}$
Control mice	13	7	0.42 ± 0.08	$1*10^{-5}$
Sham mice vs control mice				0.35

Supplementary Table 14

Statistical analysis (significant differences are highlighted in blue)

Spontaneous spiking frequency of CGIN in control, lesioned and bumetanide-treated 6-OHDA mice (cell-attached recordings)

One-way ANOVA with Fisher's LSD post-hoc test

Figure 4k

Groups	n (cells)	N (mice)	Mean ± SEM
Control mice	23	8	2.28 ± 0.33
6-OHDA mice	13	6	3.71 ± 0.35
Bumetanide-treated 6-OHDA mice	21	4	1.77 ± 0.22

Groups	n1	n2	Univariate ANOVA between groups	Post-hoc test between groups Fisher test
Control mice vs 6-OHDA mice	23	13	$4.4*10^{-4}$	0.003
6-OHDA mice vs Bumetanide-treated 6-OHDA mice	13	21		$1.1*10^{-4}$

Supplementary Table 15

Statistical analysis

Spontaneous spiking frequency of CGIN in in sham-operated and control animals (cell-attached recordings) normalized to control.

Supplementary Fig. 13b

Groups	n (cells)	N (mice)	Mean ± SEM	2 samples sample 2-tailed t-test
Sham mice	13	3	1.89 ± 0.26	0.42
Control mice			2.28 ± 0.33	

Supplementary Table 16

Statistical analysis (significant differences are highlighted in blue)

Pause-response and rebound: Spike count at different 200ms time windows taken at different times after stimulation, normalized to spike counts before train (Cell-attached recordings)

One-way ANOVA with Fisher's LSD post-hoc test

Figure 5o

Groups	n (cell)	N (mice)	Mean ± SEM
Control before train	6	4	1.00 ± 0.04
6-OHDA before train	7	4	1.00 ± 0.02
bumetanide-treated before train	8	3	1.00 ± 0.02
control 200ms aftertrain	6	4	0.044 ± 0.044
6-OHDA 200 ms aftertrain	7	4	2.28 ± 0.25
Bumetanide-treated 200 ms aftertrain	8	3	0.025 ± 0.016
control 1000ms aftertrain	6	4	2.54 ± 0.29
6-OHDA 1000 ms aftertrain	7	4	1.17 ± 0.17
Bumetanide-treated 1000 ms aftertrain	8	3	1.58 ± 0.09

Groups	n 1	n 2	Univariate ANOVA between the groups	Post-hoc test between the groups Fisher test
Control 200 ms after train vs Control before train	6	6		$9*10^{-10}$
6-OHDA 200 ms after train vs 6-OHDA before train	7	7		$8*10^{-15}$
6-OHDA 200 ms after train vs Control 200 ms after train	7	6		$6*10^{-25}$
Bumetanide treated 200 ms after train vs bumetanide treated before train	8	8		$4*10^{-10}$
Bumetanide treated 200 ms after train vs control 200ms after train	8	6		0.91
Bumetanide-treated 200 ms aftertrain vs 6-OHDA 200 ms aftertrain	8	7		$3*10^{-25}$
Control 1000 ms aftertrain vs Control before train	6	6		$2*10^{-19}$
6-OHDA 1000 ms aftertrain vs 6-OHDA before train	7	7		0.26
6-OHDA 1000 ms aftertrain vs Control 1000 ms aftertrain	7	6		$9*10^{-13}$
Bumetanide-treated 1000 ms aftertrain vs bumetanide-treated before train	8	8		$9*10^{-05}$
Bumetanide-treated 1000 ms aftertrain vs control 1000ms aftertrain	8	6		$2*10^{-07}$
Bumetanide-treated 1000 ms aftertrain vs 6-OHDA 1000 ms aftertrain	8	7	0	0.0164

Supplementary Table 17

Statistical analysis (significant differences are highlighted in blue)

Pause-response and rebound: Spike count during time windows of 200 ms taken at different times after stimulation, normalized to spike counts before train, Current-clamp recordings

One-way ANOVA with Fisher's LSD post-hoc test

Figure 5p

groups	n (cell)	N (mice)	Mean ± SEM
Control low [Cl] before train	6	4	1.074 ± 0.074
Control high [Cl] before train	3	2	0.99 ± 0.06
6-OHDA low [Cl] before train	6	4	1.01 ± 0.02
Control low [Cl] 200 ms after train	6	4	0 ± 0
Control high [Cl] 200 ms after train	3	2	0.74 ± 0.37
6-OHDA low [Cl] 200 ms after train	6	4	0 ± 0
Control low [Cl] 400 ms after train	6	4	0 ± 0
Control high [Cl] 400 ms after train	3	2	1.22 ± 0.39
6-OHDA low [Cl] 400 ms after train	6	4	0 ± 0

Groups	n1	n2	Univariate ANOVA between the groups	Post-hoc test between the groups Fisher test
Control low [Cl] before train vs control low [Cl] 200 ms after train	6	6		$9*10^{-08}$
Control high [Cl] before train vs control high [Cl] 200 ms after train	6	6		0.18
6-OHDA low [Cl] before train vs 6-OHDA low [Cl] 200 ms after train	3	3		$4*10^{-07}$
Control low [Cl] 200 ms after train vs control high [Cl] 200 ms after train	6	6		0.0016
Control low [Cl] 200 ms after train vs 6-OHDA low [Cl] 200 ms after train	6	3		1
Control low [Cl] before train vs control low [Cl] 400 ms after train	6	6		$3*10^{-08}$
Control high [Cl] before train vs control high [Cl] 400 ms after train	6	6		0.22
6-OHDA low [Cl] before train vs 6-OHDA low [Cl] 400 ms after train	3	3		$2*10^{-07}$
Control low [Cl] 400 ms after train vs control high [Cl] 400 ms after train	6	6		$2*10^{-07}$
Control low [Cl] 400 ms after train vs 6-OHDA low [Cl] 400 ms after train	6	3	1.3E-15	1

Supplementary Table 18

Statistical analysis (significant differences are highlighted in blue)

Pause-response and rebound in control and in the presence gabazine: Spike count during time windows of 400 ms taken at different times after stimulation, normalized to spike counts before train,

Cell-attached recordings

One-way ANOVA with Fisher's LSD post-hoc test

Supplementary Fig. 14b

Groups	n (cell)	N (mice)	Mean ± SEM
Control before train	6	4	1.00 ± 0.04
Gabazine before train	6	3	1.00 ± 0.02
Control 400ms aftertrain	6	4	0.044 ± 0.03
Gabazine 400 ms aftertrain	6	3	0.96 ± 0.17
Control 1000ms aftertrain	6	4	2.54 ± 0.29
Gabazine 1000 ms aftertrain	6	3	0.96 ± 0.12

Groups	n 1	n 2	Univariate ANOVA between the groups	Post-hoc test between the groups Fisher test
Control 400 ms after train vs control before train	6	6	0	$3*10^{-10}$
Gabazine 400 ms after train vs gabazine before train	6	6		0.73
Gabazine 400 ms after train vs control 400 ms after train	6	6		$7*10^{-9}$
Control 1000 ms aftertrain vs control before train	6	6		$1.4*10^{-14}$
Gabazine 1000 ms aftertrain vs gabazine before train	6	6		0.78
Gabazine 1000 ms aftertrain vs control 1000 ms aftertrain	6	6		$8*10^{-12}$

Supplementary Table 19

Statistical analysis (significant differences are highlighted in blue)

Effects of nicotinic receptors antagonists on spontaneous IPSCs frequency (normalized to control) recorded on SPN neurons (total and "giant")

Figure 6c

**Groups**	n (cells)	N (mice)	Mean ± SEM	Paired sample 2-tailed t-test
sIPSCs control	7	6	1 ± 0	0.0049
sIPSCs + nicotinic receptor antagonists	7	6	0.29 ± 0.16	
Giant IPSCs control	7	6	1 ± 0	$1*10^{-7}$
Giant IPSCs + nicotinic receptor antagonists	7	6	0.052 ± 0.033	

Supplementary Table 20

Effects of bumetanide on spontaneous IPSCs frequency in SPN neurons normalized to control

Figure 6d

**Groups**	n (cells)	N (mice)	Mean ± SEM	Paired sample 2-tailed t-test
sIPSC control	7	6	1 ± 0	$2*10^{-4}$
sIPSC bumetanide	7	6	0.29 ± 0.09	
Giant IPSC control	7	6	1 ± 0	$6*10^{-7}$
Giant IPSC bumetanide	7	6	0.05 ± 0.04	

Supplementary Table 21

Statistical analysis (significant differences are highlighted in blue)

Time to traverse roller

Figure 6e

**Groups**	N (mice)	Mean ± SEM (s)	2-sample 2-tailed-t-test
control mice	9	6.40 ± 0.54	P=0.03
6-OHDA mice (first measurement)	7	8.19 ± 0.42	

Groups	N (mice)	Mean ± SEM (s)	Paired 2-tailed-t-test
6-OHDA mice (second measurement)	7	8.24 ± 0.47	P=0.03
bumetanide-treated 6-OHDA mice	7	6.31 ± 0.61	

Supplementary Table 22

Statistical analysis (significant differences are highlighted in blue)

Pole test

Score. One-way ANOVA Fisher's with LSD post-hoc test

Figure 6f

Groups	N (mice)	Mean ± SEM
Control	7	4.80 ± 0.86
6-OHDA	9	8.10 ± 0.71
Bumetanide-treated 6-OHDA	8	5.11 ± 0.76

Groups	N1	N2	Univariate ANOVA between the groups	Post-hoc test between the groups Fisher test
Control vs 6-OHDA	7	9	0.00973	0.007
6-OHDA vs bumetanide-treated 6-OHDA	9	8		0.79
Control vs bumetanide-treated 6-OHDA	7	8		0.01

Supplementary Table 23

Taqman® primers used for qPCR

Target Gene	Taqman® gene expression assay ID	fluorescence	Excitation/Emission
Lhx8/7	Mm00802919_m1	Fam-MGB	494 -518 nm
Lhx6	Mm01333348_m1	Fam-MGB	494-518 nm
ChAT	Mm01221880_m1	Fam-MGB	494-518 nm
Gad2	Mm00484623_m1	Fam-MGB	494-518 nm
HPRT	Mm03024075_m1	VIC-MGB	538-554 nm

Supplementary figures

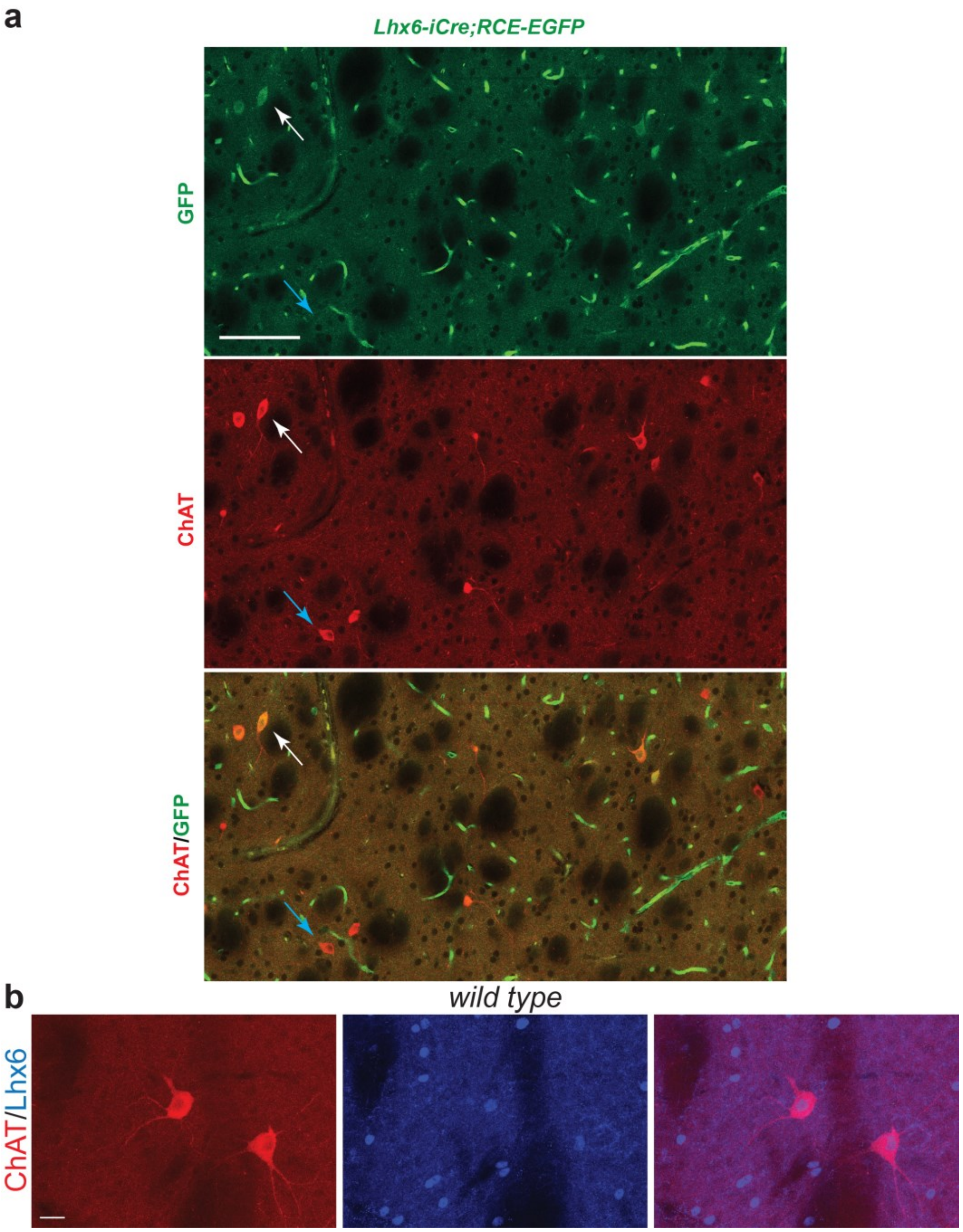

Supplementary Figure 1 Co-expression of ChAT and EGFP or Lhx6 in a subpopulation of cholinergic interneurons. a EGFP (green)/ChAT (red) co-immunostaining of coronal striatal slices from Lhx6-iCre;RCE-EGFP mice showing expression of EGFP in a subset of cholinergic cells. White arrows: $EGFP^{+}$ $ChAT^{+}$ cells, Blue arrows: $EGFP^{-}$ $ChAT^{+}$ cells. Scale bar: 100 μm. **b** Co-immunostaining of ChAT (red) and Lhx6 (blue) of coronal striatal slices from wild type mice showing expression of Lhx6 in a subset of cholinergic cells. Scale bar: 20 μm.

Lhx6-iCre;RCE-EGFP mice
CGIN

a

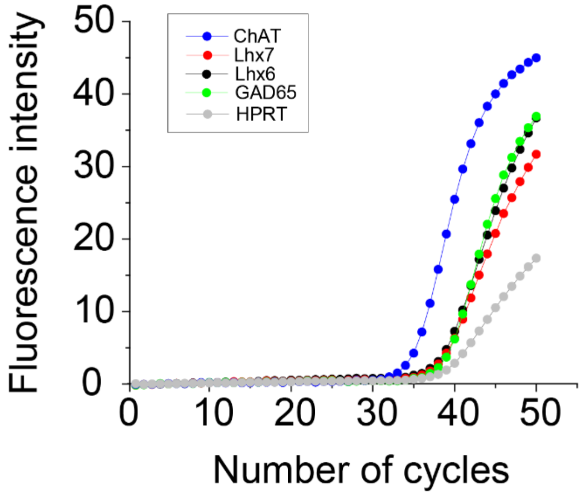

b

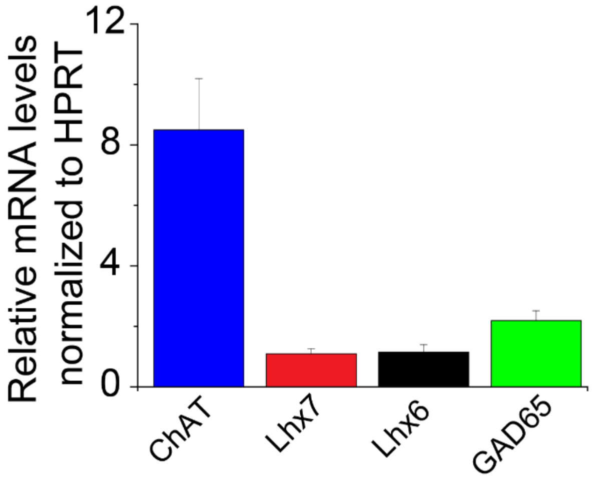

Supplementary Figure 2 Real-time single cell RT-qPCR. **a** Amplification plots for ChAT, Lhx6, Lhx7, GAD65 and HPRT mRNAs for a representative single EGFP+ cholinergic interneuron (Lhx6-iCre;RCE-EGFP mouse). Relative fluorescence intensities (arbitrary units) plotted against PCR cycle numbers on a linear scale. **b** Mean expression of ChAT (n = 11), Lhx6 (n = 5), Lhx7 (n = 11) and GAD65 (n = 10) mRNAs normalized to the expression of hypoxanthine phosphoribosyltransferase (HPRT). The mean values of Lhx6 or GAD65 mRNAs were calculated from the subset of ChAT mRNA expressing cells that also expressed Lhx6 or GAD65 mRNAs.

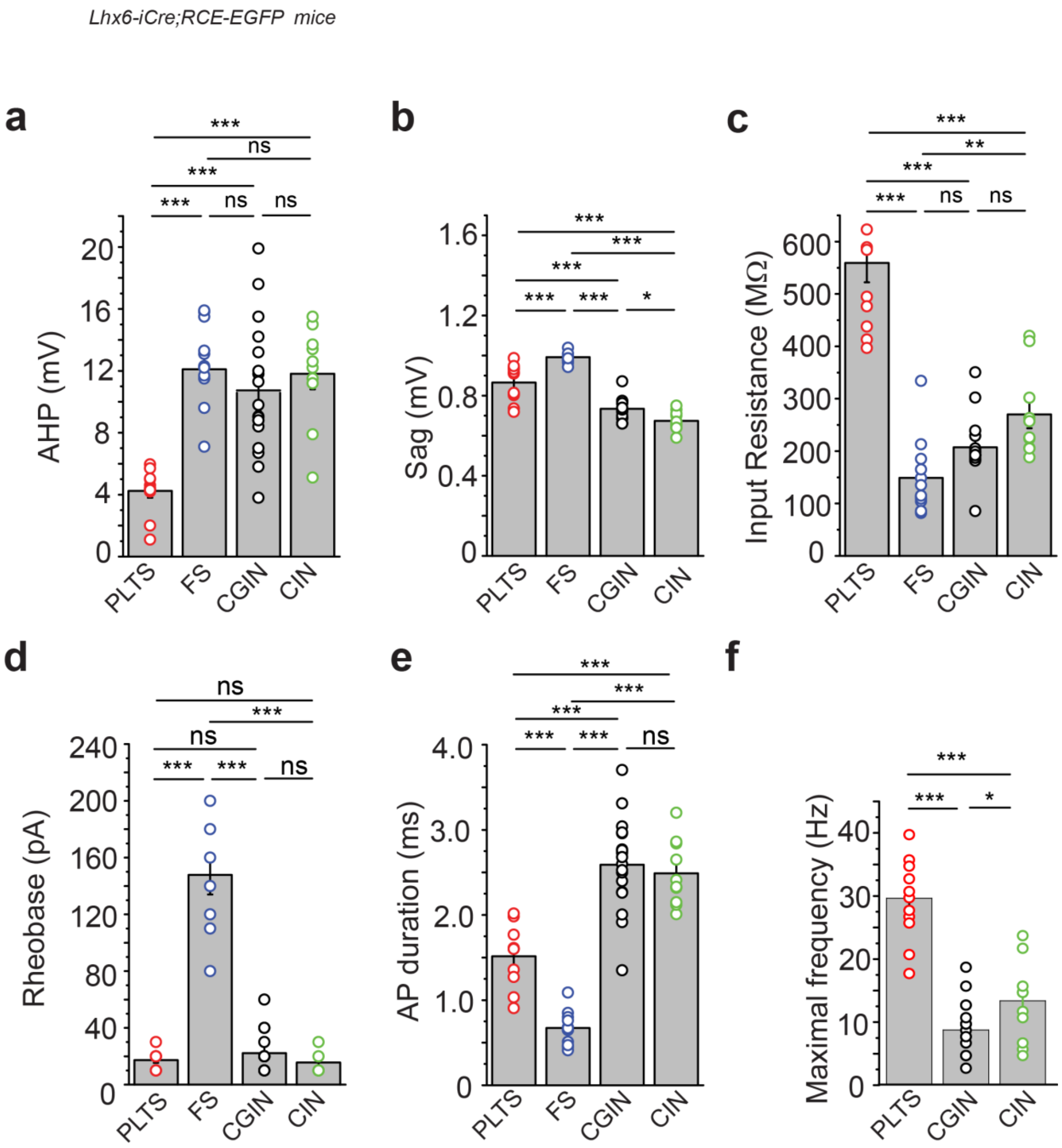

Supplementary Figure 3 Similarities and differences of intrinsic membrane properties of striatal interneurons. Quantification of the main intrinsic membrane properties of PLTS, FS, CGIN and CIN interneurons (Lhx6-iCre;RCE-EGFP mice). AHP: afterhyperpolarization, AP: action potential. All means± s.e.m. All data sets were analyzed using one-way ANOVA followed by Fisher's Least Significant Difference (LSD) post hoc test. See Supplementary Table 3 for statistics; $^{*}P<0.05$, $^{**}P<0.01$, $^{***}P<0.001$. ns, not significant.

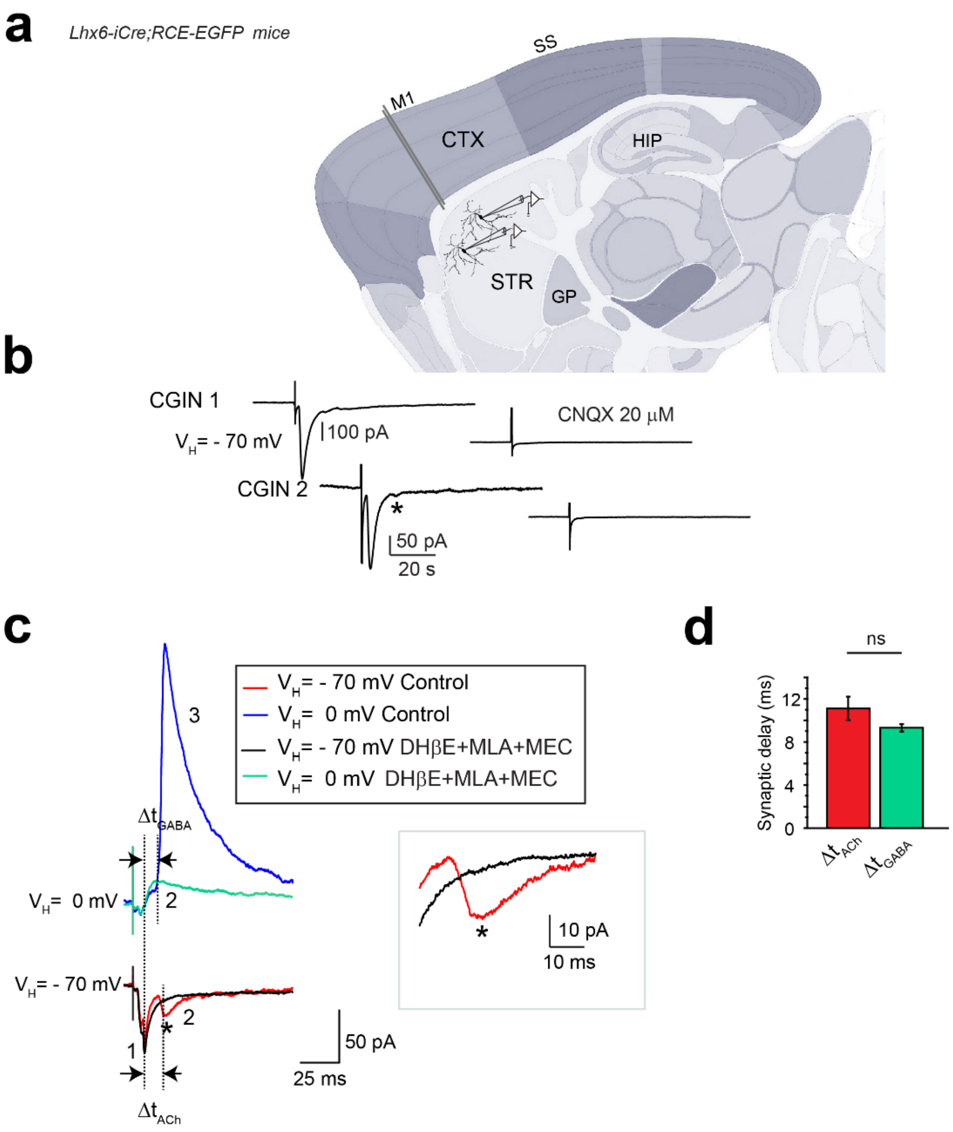

Supplementary Figure 4 Cortical stimulation evokes a glutamatergic PSC followed by nicotinic and GABAergic PSCs in CGINs (Lhx6-iCre;RCE-EGFP mice). a Experimental setup. Scheme of a sagittal slice showing the location of the extracellular stimulating electrode in the corpus callosum and of the recorded CGIN. CTX: cortex, M1: motor cortex, SS: somatosensory cortex, STR: striatum, HIP: hippocampus, GP: Globus Pallidus. **b** Cortical stimulation reliably evoked a glutamatergic EPSC in CGIN at V_H = - 70 mV, which was blocked by CNQX (20 µM). **c** Further analysis showed that stimulation generated in CGIN at V_H = - 70 mV a large glutamatergic PSC, followed by a second nicotinic receptor mediated PSC (asterisk) with delay (insert: enlarged scale). In addition, the stimulation evoked at the reversal potential of glutamatergic currents (V_H = 0) a composite (early and late) GABAergic PSC. Nicotinic receptor antagonists abolished the nicotinic and late GABAergic PSCs, but not the glutamatergic and early GABAergic PSCs (n = 3). **d** Delays between the glutamatergic PSC peak and the nicotinic or the early GABAergic PSC peaks were similar.
All means±s.e.m. **d** Significance was determined by two-tailed, unpaired Student's t-test; n=5; ns, not significant.

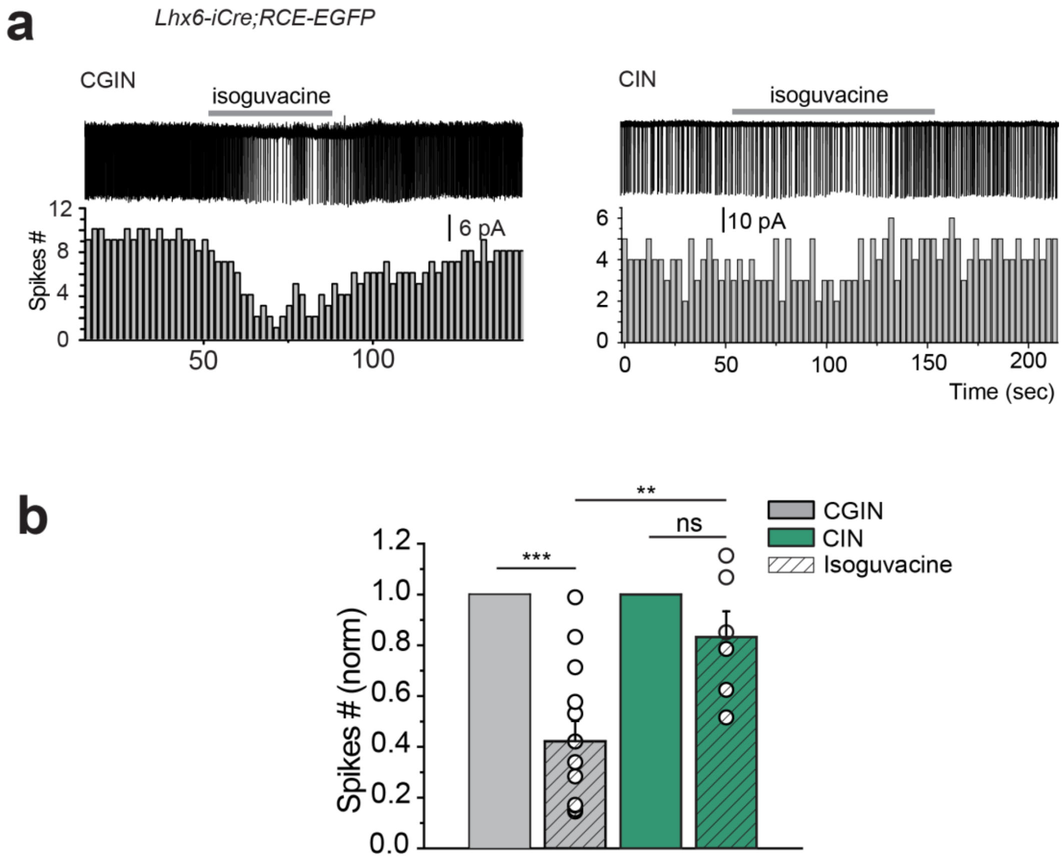

Supplementary Figure 5 GABA inhibits ongoing activity in CGINs not CINs. a Representative cell-attached recording of the inhibitory effect of applications of isoguvacine (10 µM) on the spontaneous activity of a CGIN and CIN in control. **b** Corresponding mean values of isoguvacine maximal effect and comparison with values in CGINs (the same set of data as in Figure 4 from control mice). All means±s.e.m. **d** Data sets were analyzed using one-way ANOVA followed by Fisher's Least Significant Difference (LSD) post hoc test. See Supplementary Table 5 for statistics; **P<0.01, ***P<0.001. ns, not significant.

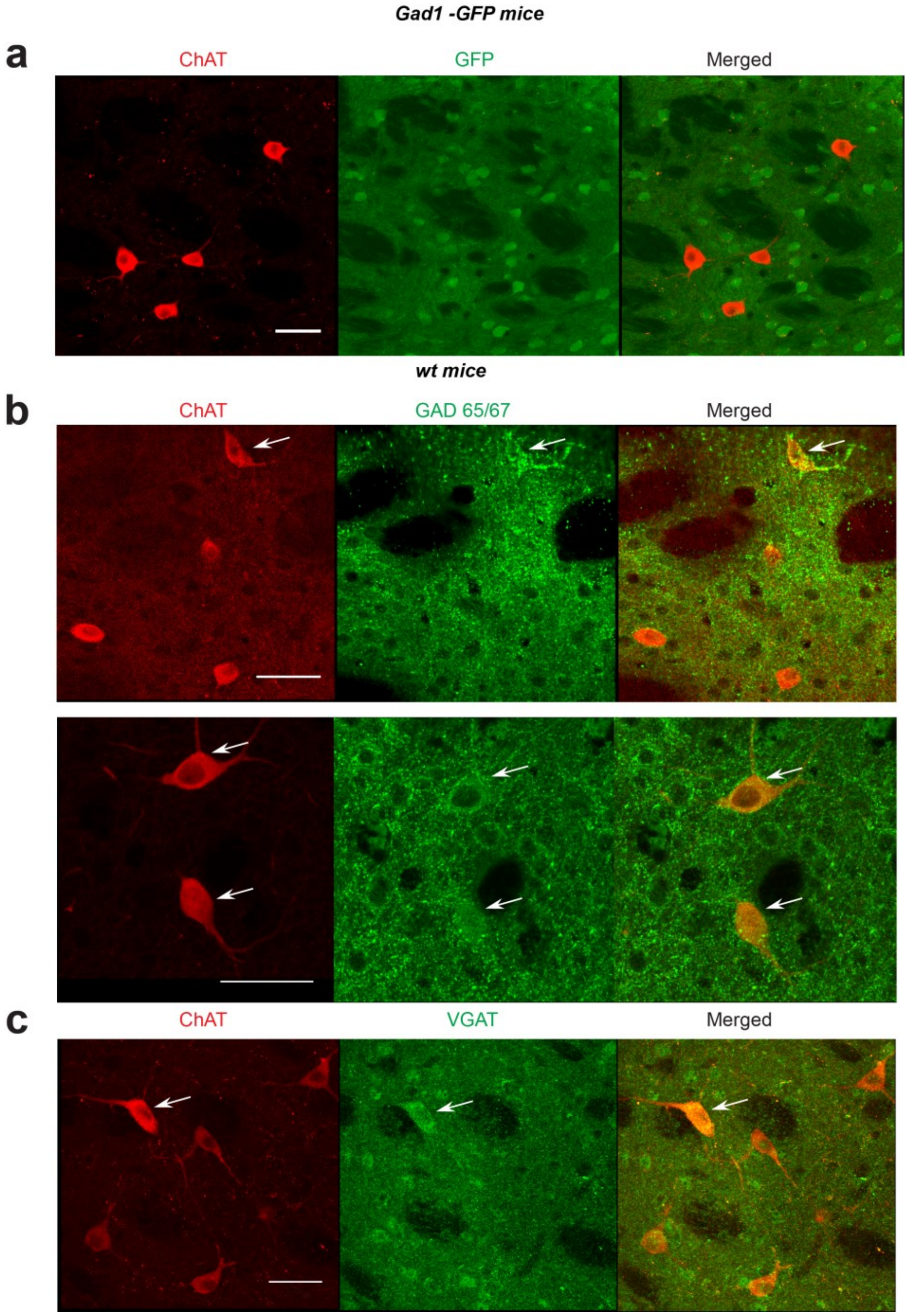

Supplementary Figure 6 A subpopulation of immunopositive ChAT cells in the dorsolateral striatum expresses GABAergic markers.

a ChAT (red), GFP (green) co-immunostaining of coronal striatal slices from Gad1-GFP mice showing the absence of GFP expression in ChAT+ cells somata. Scale bar: 40 µm. **b** ChAT (red), GAD65/67 (green) co-immunostaining of coronal striatal slices from wt mice showing expression of GAD65/67 in cholinergic neurons. Scale bar: 40 µm. **c** ChAT (red), VGAT (green) co-immunostaining of coronal striatal slices from wt mice showing co-expression of VGAT and ChAT in a subset of cholinergic neurons. Scale bar: 40 µm. See Supplementary Table 6 for statistics.

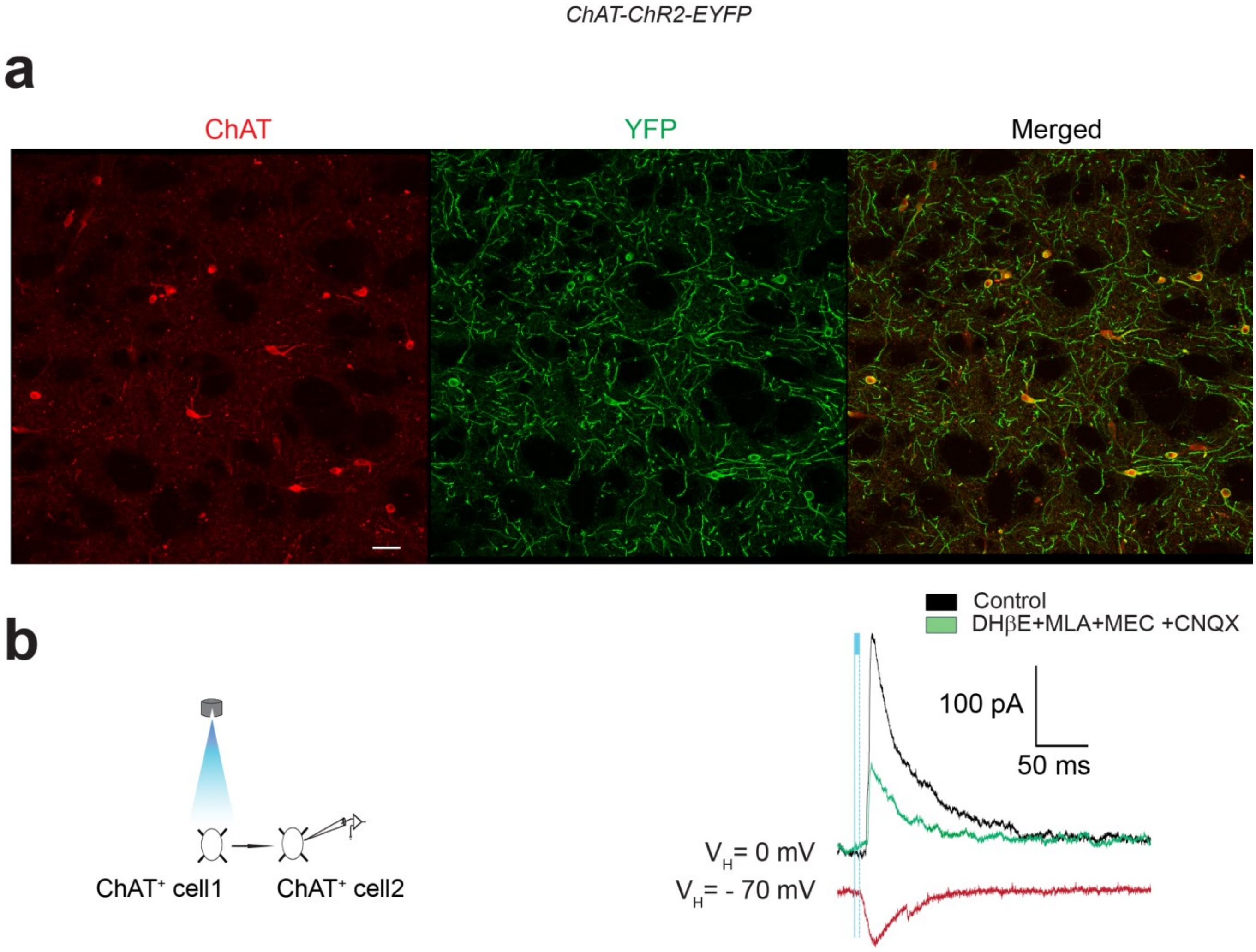

Supplementary Figure 7 CGINs co-release ACh and GABA in striatum from control ChAT-ChR2-YFP mice. a 98% of cholinergic interneurons express YFP. ChAT (red), YFP (green) co-immunostaining of coronal slices from ChAT-ChR2-YFP mice showing co-expression of ChAT and ChR2-YFP. Scale bar: 40μm. See supplementary Table 8 for statistics. **b** Outward (black) and inward (red) PSCs evoked in postsynaptic whole-cell recorded $ChAT^+$ cell2 in response to optogenetic stimulation of presynaptic $ChAT^+$ cell1 in control ACSF. Nicotinic receptor (MEC (10 μM), MLA (0.1 μM), DHβE (10 μM) and AMPA receptor (CNQX, 20 μM) antagonists blocked the polysynaptic but not the monosynaptic outward GABAergic PSC (green).

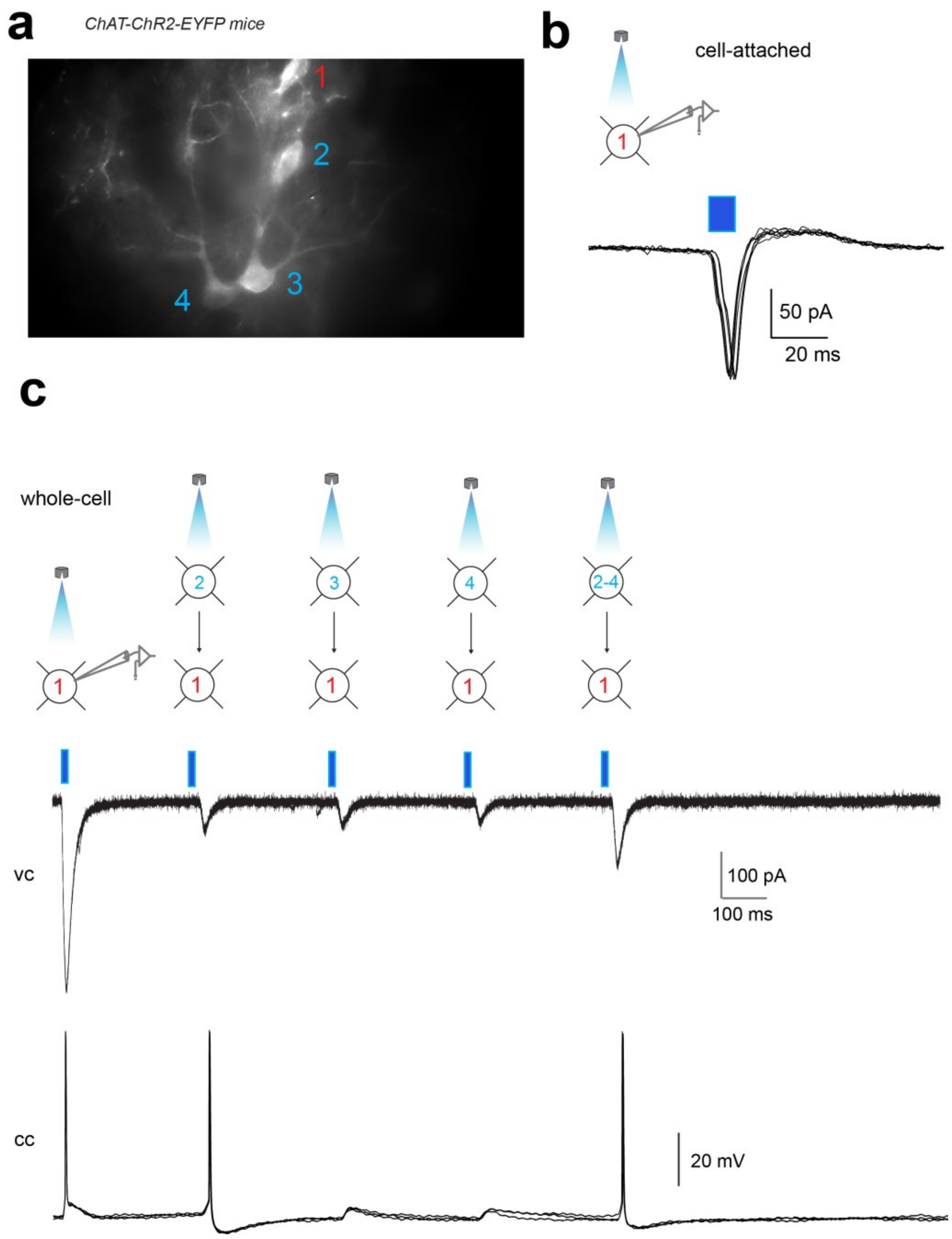

Supplementary Figure 8 High-resolution optical control of spatiotemporal neuronal activity of cholinergic neurons using a digital micro-mirror device (DMD). a Fluorescent image of YFP/ChAT/channelrhodopsin-2 (ChR2) expressing cells in a sagittal slice from a ChAT-ChR2-YFP mouse. **b** ChR2 activation by a brief light pulse (490 nm, 10 ms) reliably triggered an action potential in a ChR2-expressing neuron with millisecond temporal precision (cell-attached configuration). **c** Somatic stimulation of single neurons (1, 2, 3 or 4) or synchronous somatic stimulation of neurons 2 to 4, that all expressed ChR2, reliably evoked an EPSC, EPSP or action potential in cell 1. vc: whole-cell voltage clamp mode; cc: whole cell current clamp mode.

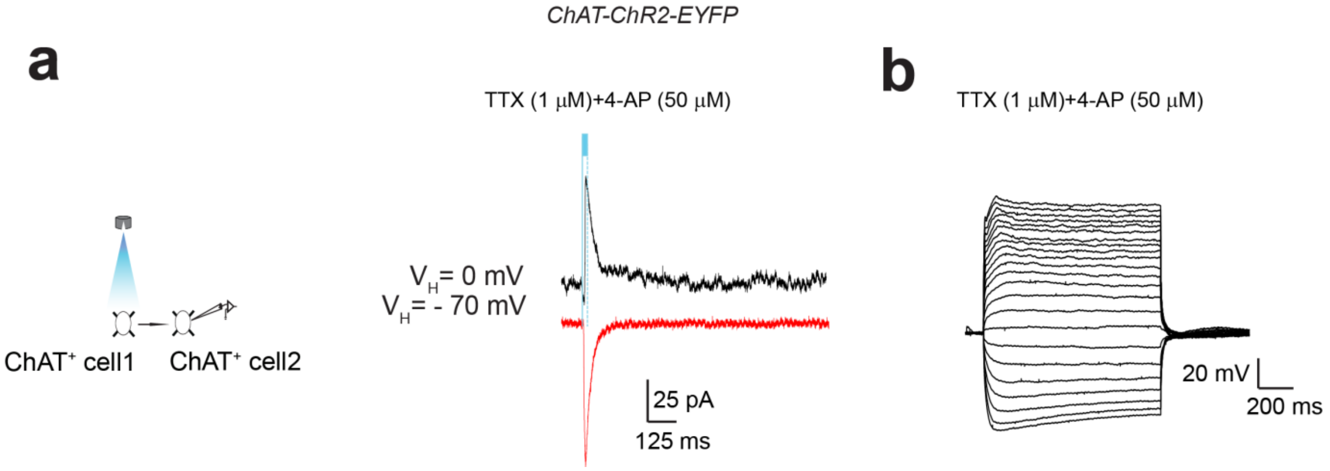

Supplementary Figure 9 Cholinergic cell co-releases ACh and GABA in control ChAT-ChR2-YFP mice in condition of suppressed ongoing activity and enhanced release probability.
Monosynaptic GABAergic PSC evoked in postsynaptic whole cell recorded cholinergic cell 2 in response to optogenetic stimulation of presynaptic ChAT$^+$ cell1 in the presence of TTX (1 μM) and 4-AP (50 μM). (Right) voltage responses of ChAT$^+$ cell2 to current steps, showing that action potentials are fully blocked in these conditions.

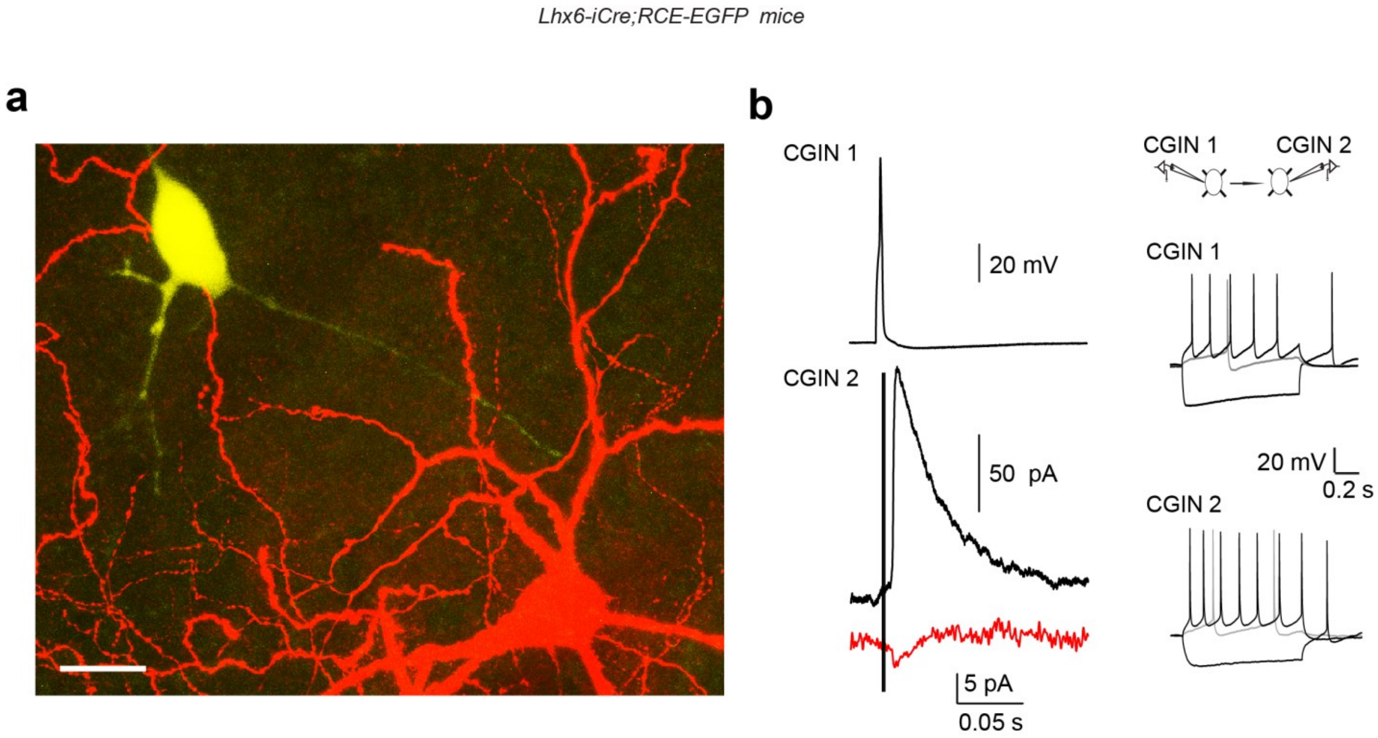

Supplementary Figure 10 CGINs co-release ACh and GABA in control Lhx6-iCre;RCE-EGFP mouse. a Representative image of two identified biocytin-filled (red, CGIN1) and Lucifer Yellow –labeled (yellow, CGIN2) monosynaptically-connected CGINs. **b** (left) Outward (GABAergic) and inward (cholinergic) PSCs evoked in postsynaptic CGIN2 (bottom) in response to a presynaptic spike generated in CGIN1 (top); (right) firing patterns of CGIN1 and CGIN2. Scale bar: 20µm.

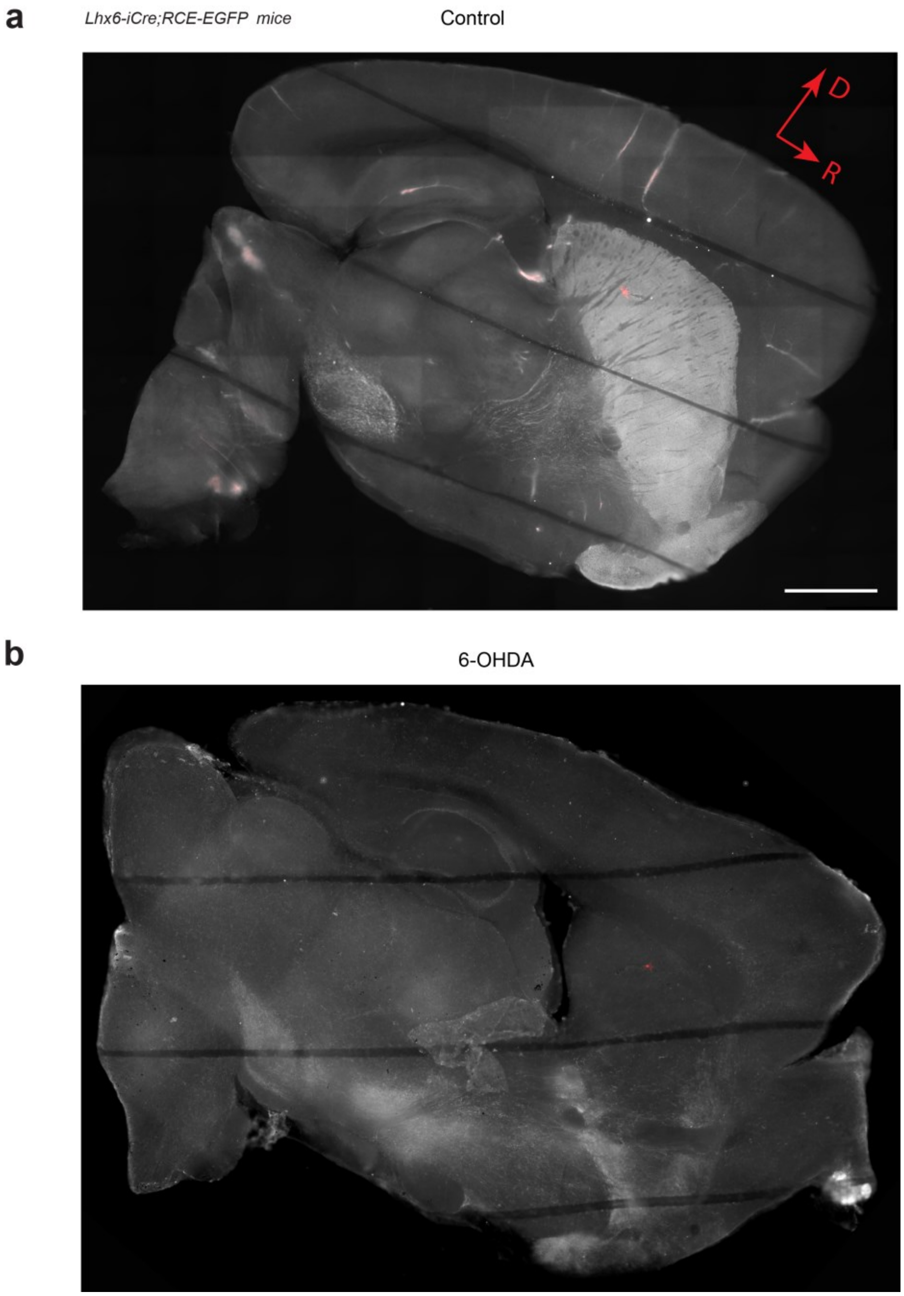

Supplementary Figure 11 TH immunolabeling (white) and biocytin-filled neuron (red). Sagittal slices from control (a) and 6-OHDA-treated (b) Lhx6-iCre;RCE-EGFP mice. Note the absence of TH labeling in the dorsal striatum of the 6-OHDA-treated mouse. Scale bar: 1 mm. D: dorsal; R: rostral.

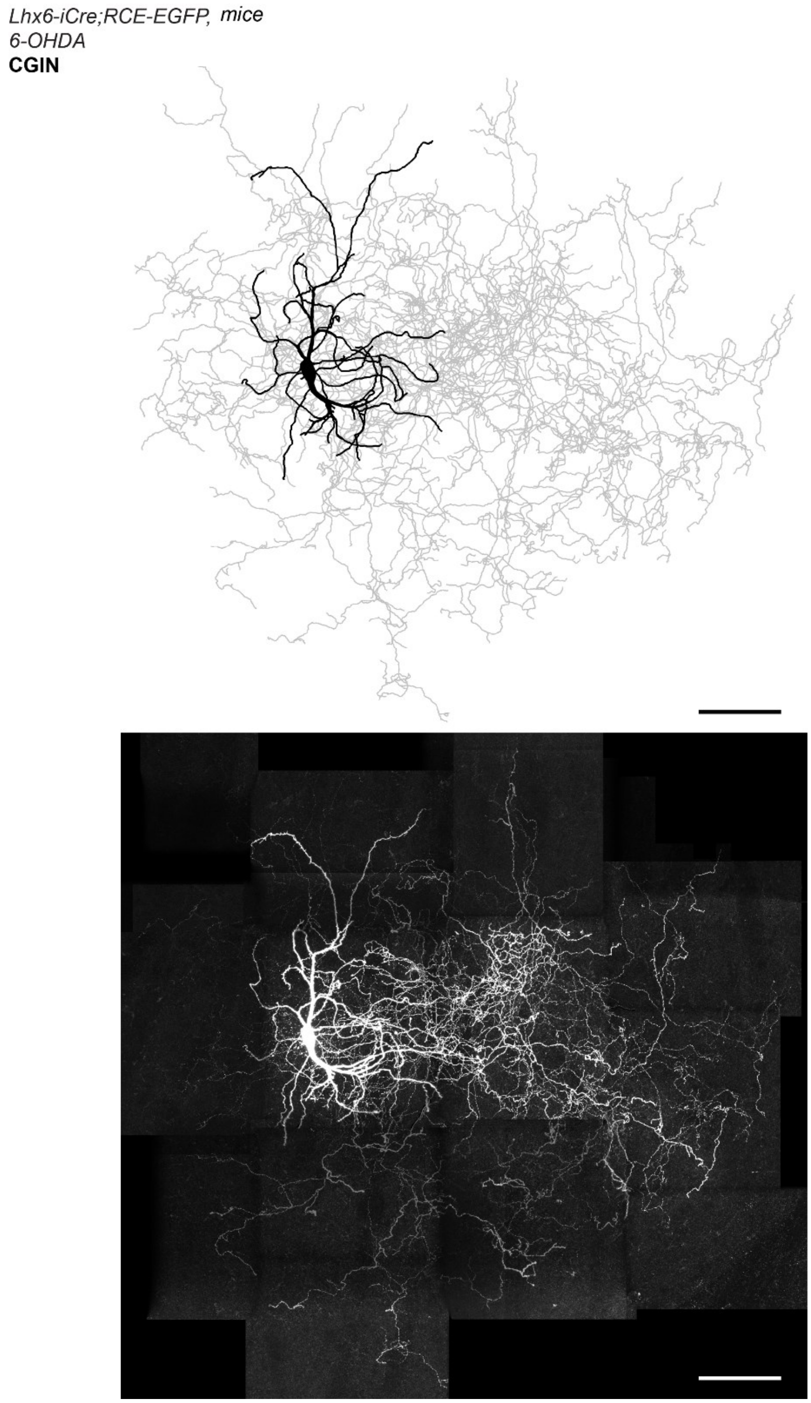

Supplementary Figure 12 Morphology of a CGIN from a 6-OHDA-treated Lhx6-iCre;RCE-EGFP mouse. (Top) Reconstruction and (bottom) confocal microphotography of the dendritic tree and the large axonal arbor. Scale bars: 100 µm.

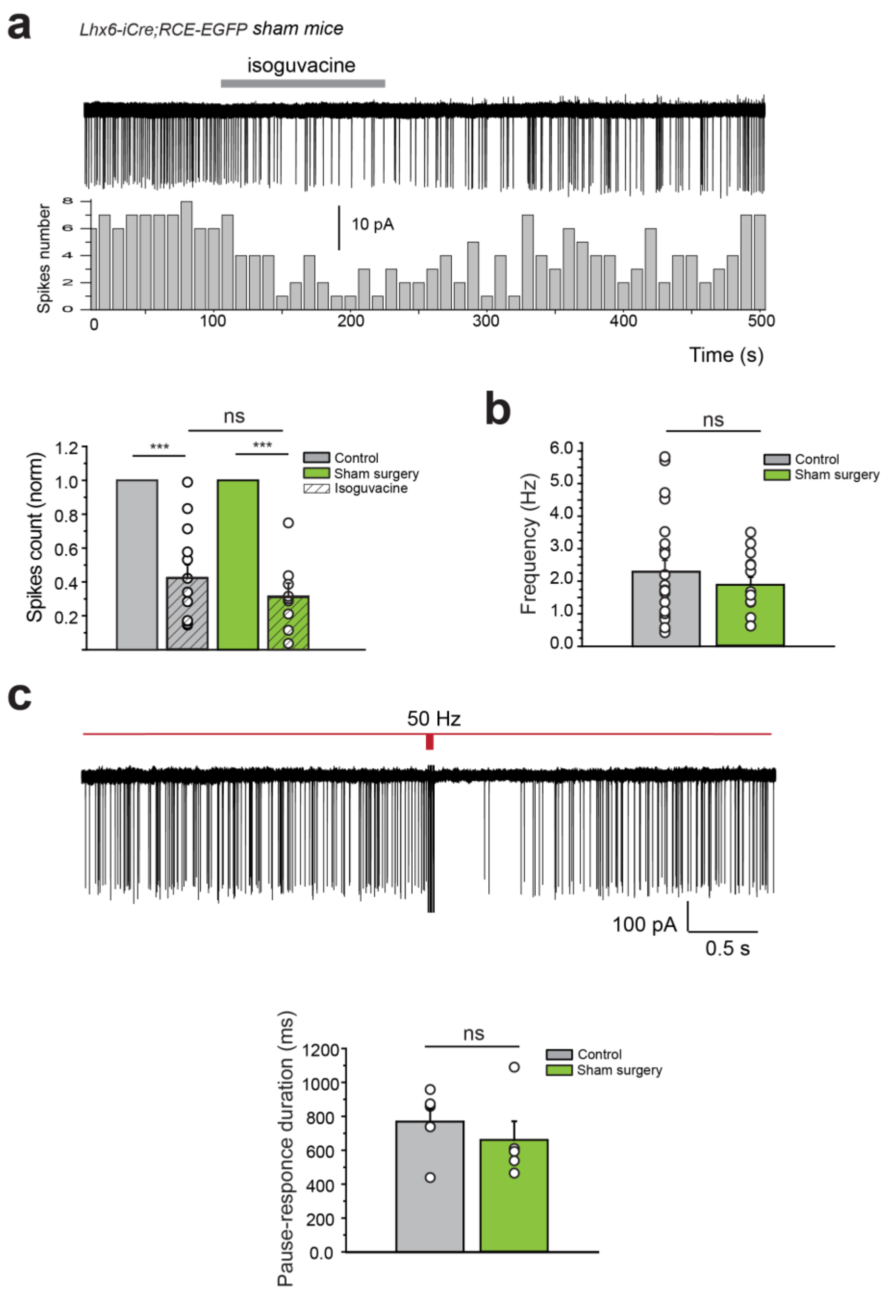

Supplementary Figure 13 Sham surgery did not affect GABA inhibition or pause response of CGINs (Lhx6-iCre;RCE-EGFP mice). **a** (top) Inhibitory effects of isoguvacine (10 μM) on spontaneous activity of a CGIN from a sham-operated mouse. (Bottom) Mean values of isoguvacine maximal effects in sham operated and control mice. **b** Spontaneous CGINs instantaneous spike frequency in control and sham-operated mice before isoguvacine application. **c** (Top) Representative pause response evoked by cortical stimulation (cell-attached recording). Similar durations of the pause response in CGINs from control and sham-operated mice (bottom). All means±s.e.m. **a** Data sets were analyzed using one-way ANOVA followed by Fisher's Least Significant Difference (LSD) post hoc test. **b, c** Significance was determined by two-tailed, unpaired Student's t-test; See Supplementary Tables 13, 15 for statistics; ***$P<0.001$. ns, not significant.

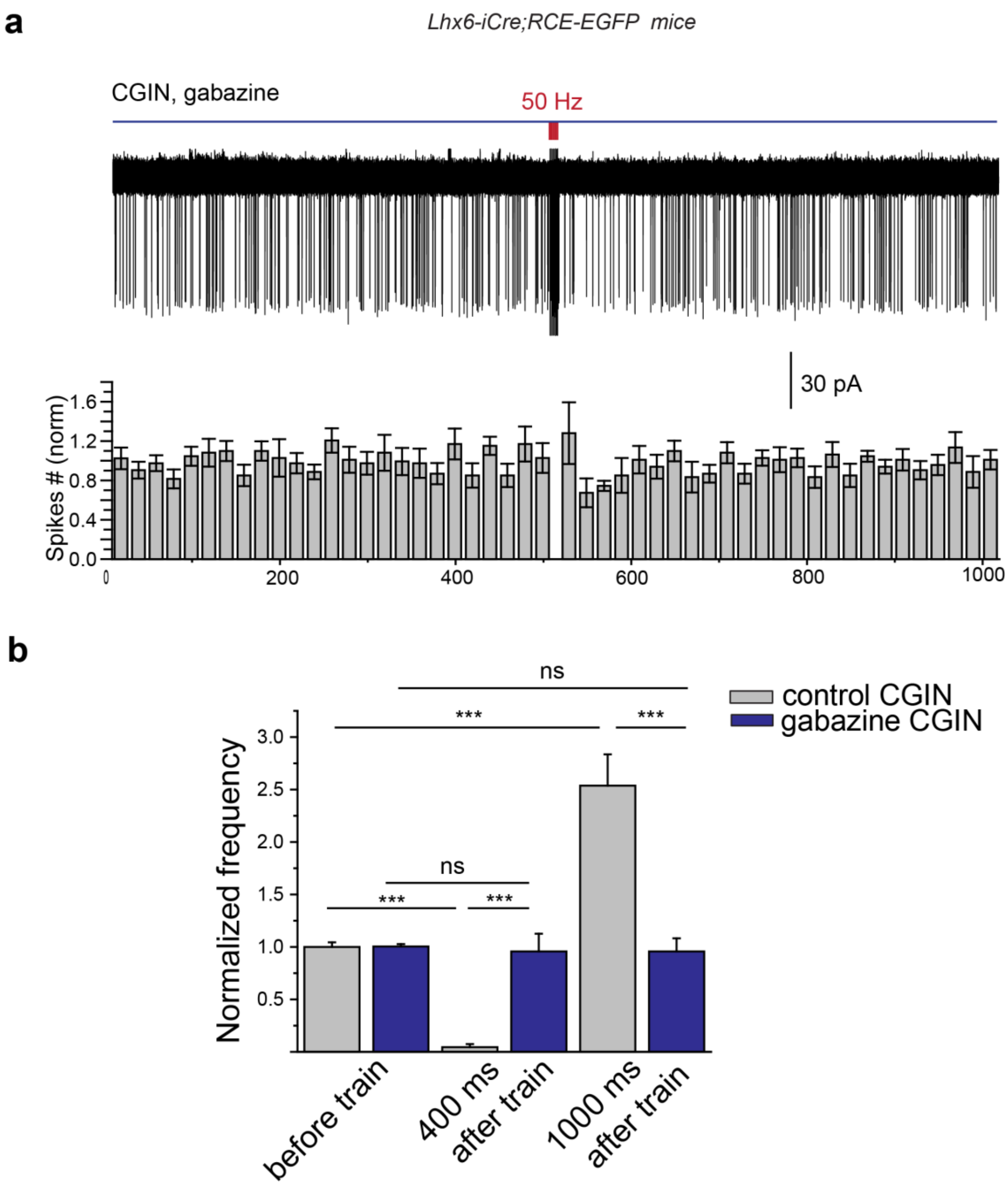

Supplementary Figure 14 GABAergic signaling is a critical determinant of cortically-evoked pause-rebound response in CGINs. (**a**, Top) Stimulation protocol. Representative several superimposed consecutive CGINs responses to cortical stimulation (cell-attached recording) and (**a**, Bottom) mean frequency histograms, in the continuous presence of 10 μM of gabazine. **b** Mean number of spikes during time windows after train stimulation (0-400 ms, 800-1000 ms) normalized to spikes counts before train in control (the same set as shown in **Figs 1,5** in the presence of gabazine. All means±s.e.m. **b** Data sets were analyzed using one-way ANOVA followed by Fisher's Least Significant Difference (LSD) post hoc test. See Supplementary Table 18 for statistics; ***P<0.001. ns, not significant.

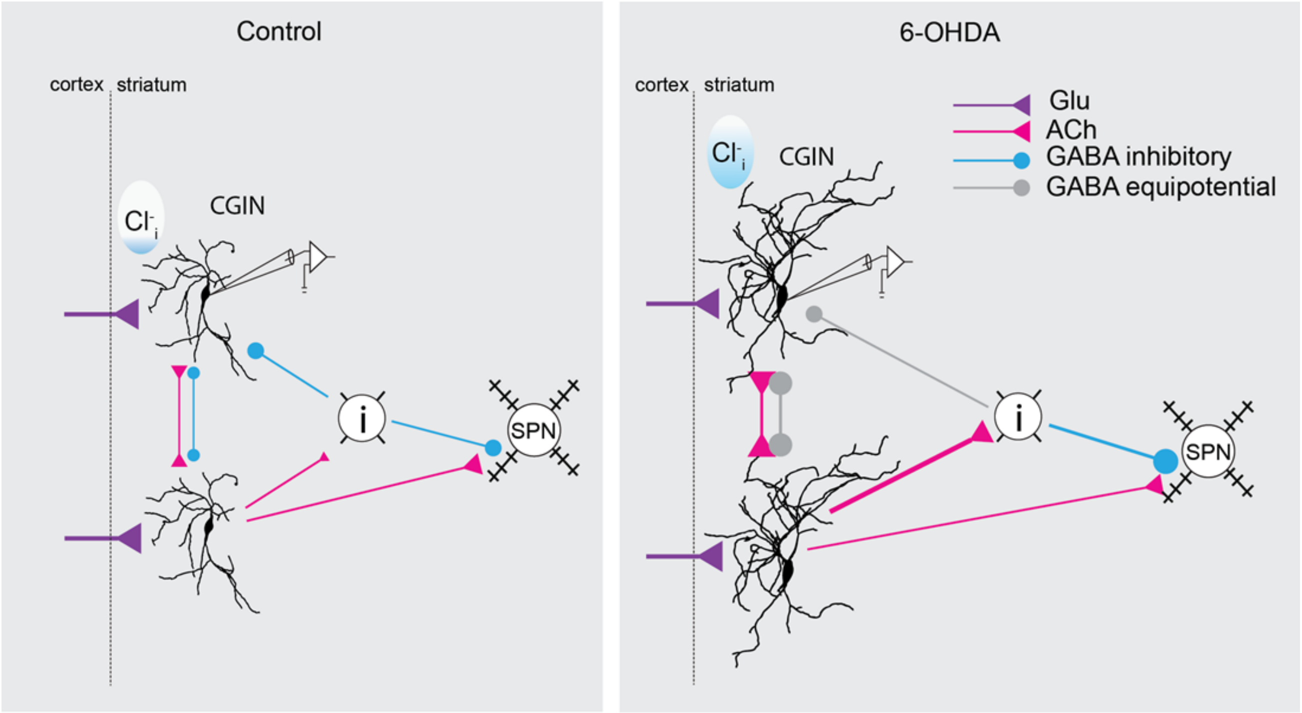

Supplementary Figure 15 Schematic representation of the putative striatal network activated by cortical stimulation in control (left) and 6-OHDA-treated mice (right). Cortical glutamatergic input triggers a direct excitatory PSC in the recorded CGIN. Activation of other CGINs monosynaptically connected to the recorded one triggers a delayed recurrent dual ACh/GABA PSC in the recorded CGIN. In control conditions, GABAergic inhibition is efficient due to "low" $[Cl^-]_i$ levels in CGINs. In contrast, in 6-OHDA-treated mice, $[Cl^-]_i$ is high in CGINs, GABAergic inhibition is abolished, leading to increased cholinergic excitation in CGINs network. This also impacts other non-cholinergic striatal GABAergic interneurons via nicotinic receptor activation leading to neuronal and network hyperactivity manifested notably by the giant GABAergic PSCs in SPNs[1,2].

Supplementary References

1. English, D. F. et al. GABAergic circuits mediate the reinforcement-related signals of striatal cholinergic interneurons. *Nat. Neurosci.* 15, 123–130 (2012).
2. Dehorter, N. *et al.* Subthalamic Lesion or Levodopa Treatment Rescues Giant GABAergic Currents of PINK1-Deficient Striatum. *Journal of Neuroscience* **32,** 18047–18053 (2012).
