# Supplementary material for: GABAergic inhibition in dual-transmission cholinergic and GABAergic striatal interneurons is abolished in Parkinson disease
Source: Nat Commun. 2018 Apr 12;9:1422. doi: 10.1038/s41467-018-03802-y (PMC5897332; doi:10.1038/s41467-018-03802-y)
Supplement: Supplementary file 3 — Description of Additional Supplementary Files [file 41467_2018_3802_MOESM3_ESM.pdf]

Description of Additional Supplementary Files

File Name: Supplementary Movie 1
Description: Quantitative assessment of CGINs/CINs ratio in dorsolateral striatum using iDISCO approach. iDISCO experimental design: segmentation of the striatum and extraction of a region of interest (ROI, dorsolateral striatum) with IMARIS software. ChAT staining (red) in representative dorsolateral striatum from Lhx6-iCre; RCE-EGFP mouse

File Name: Supplementary Movie 2
Description: Pole test. Control mouse

File Name: Supplementary Movie 3
Description: Pole test. 6-OHDA-treated mouse

File Name: Supplementary Movie 4
Description: Pole test. 6-OHDA-treated mouse after bumetanide treatment

File Name: Supplementary Movie 5
Description: Roller test. Control mouse

File Name: Supplementary Movie 6
Description: Roller test. 6-OHDA-treated mouse

File Name: Supplementary Movie 7
Description: Roller test. The same 6-OHDA-treated mouse as in Movie 6 after bumetanide treatment
